# Long term GP opinions and involvement after a consultation-liaison intervention for mental health problems

**DOI:** 10.1186/1471-2296-9-41

**Published:** 2008-07-02

**Authors:** Nadia Younès, Christine Passerieux, Marie-Christine Hardy-Bayle, Bruno Falissard, Isabelle Gasquet

**Affiliations:** 1 Academic Unit of Psychiatry, Versailles Hospital, Le Chesnay, France; 2 INSERM U669, Hôpital Cochin, AP-HP, Paris, France; 3 University of Versailles St Quentin en Yvelines (Paris Ile de France Ouest), Guyancourt, France; 4 University Paris-Sud, Paris, France; 5 University of Paris 5, Paris, France; 6 Direction of Medical Politicy, Paris, France

## Abstract

**Background:**

Shared Mental Health care between Psychiatry and Primary care has been developed to improve the care of common mental health problems but has not hitherto been adequately evaluated. The present study evaluated a consultation-liaison intervention with two objectives: to explore long-term GP opinions (relating to impact on their management and on patient medical outcome) and to determine the secondary referral rate, after a sufficient time lapse following the intervention to reflect a "real-world" primary care setting.

**Methods:**

All the 139 collaborating GPs (response rate: 84.9%) were invited two years after the intervention to complete a retrospective telephone survey for each patient (181 patients; response rate: 69.6%).

**Results:**

91.2% of GPs evaluated effects as positive for primary care management (mainly as support) and 58.9% noted positive effects for patient medical outcome. Two years post-intervention, management was shared care for 79.7% of patients (the GP as the psychiatric care provider) and care by a psychiatrist for 20.3% patients. Secondary referral occurred finally in 44.2% of cases.

**Conclusion:**

The intervention supported GP partners in their management of patients with common mental health problems. Further studies are required on the appropriateness of the care provider.

## Background

The care of common mental health problems, mainly provided in primary care, is known to be insufficient and to represent a major public health challenge. Collaborative care between Psychiatry and Primary care has been developed in several countries to address this problem [1,2]. Evaluation of this kind of care is necessary. M Craven and R Bland reviewed the changing trends in collaborative care research: early studies were descriptive, and concerned with the impact on system outcomes. Then they focused on patient-level outcomes and quality improvement initiatives. Recently the ability of research-based programs to be translated into "real-world" settings has been examined [3]. Existing studies of shared mental health care considering a long-term outcome (more than a year) have focused on the patient [4]. They did not explore long-term GP opinions and long-term care delivery.

Two objectives were thus set out for the present study. First, to provide data on GP's opinions on the impact of the consultation-liaison system (after sufficient time had passed to enable a more "objectively" based view of the consultation system outcome), on their management of the patients they referred (rather than transferred) to the collaborative system, and on patient medical outcome. Second, to determine how frequently the family physician remained the primary care provider, alone or in conjunction with a mental health worker, after the shared mental health care intervention, with a sufficient time lapse following the intervention to reflect the real-world care delivery [2,5,6].

## Methods

A retrospective study was implemented in September 2004, i.e three years after the start of the intervention.

The intervention involves a specialist consultation center intended for GPs in the geographic area ("South Yvelines" area to the west of Paris, 650 000 inhabitants, 492 GPs) which is located in the biggest general hospital, less stigmatized than psychiatric hospitals [7]. Primary care physicians are invited to seek support for patients whom they consider pose a problem, without transferring the main responsibility to specialty care. The staffs is composed of Mental health professionals: two full-time equivalent psychiatric nurses, one full-time equivalent psychiatrist and one full-time equivalent psychologist. Each year since the start of the system 500 requests have been recorded. Patients referred to the consultation-liaison system by GPs are the main candidates, but direct requests from patients are also accepted if patients agree to a phone contact with their GP. A psychiatric nurse receives requests, comes into contact with the patient and systematically calls the GP to know his/her expectations about the intervention. Several responses are possible: direct orientation (specialized management for substance abuse or emergencies) after the phone contact without a specialized consultation (22%) or, mainly, psychiatrist or psychologist consultations for advice or joint interventions (78%). The objective of the intervention is to reach a decision on the way in which the patients should be catered for on the basis of the relationship with the GP, the expectations of both patient and GP, and the opinion of the consultant. No standard recommendations are used, except the required contacts with the GP subject to patient agreement (before the response, after consultation and a mail response). The intervention consisted for 41% of the patients in one consultation, for 31% in two or three consultations, and for 28% in more than three consultations. After the intervention, 30.0% of the patients were referred to a mental health facility and 70.0% returned to their GP. Every patient was informed before of the first consultation of the experimental care he will receive and gave written consent to researches on the intervention. The ethic committee of Paris Ile de France Ouest University gave approval for the study.

### Population

The population was composed of the GPs (n = 139), who, as the main psychiatric care providers, received patients returned to them after the consultation-liaison intervention (70.0% of the patients, since 30.0% were referred to the specialized care system). For these patients, GPs were invited to complete a retrospective telephone survey (260 patients expected), two years on average after the intervention (mean = 23.9 months, sd = 8.9, min = 6, max = 40). 3 attempts to contact GPs were fixed.

### Data collected

The telephone questionnaire, requiring 10 minutes to complete, measured the way in which collaborative relationships developed between GPs and psychiatrists, GPs' opinions on the impact of the intervention, and continuing management after the intervention. Closed items were included for each type of data, completed with open items to explore referral reasons and GPs' opinions about the interventions.

### Data analysis

Analyses were performed with SAS 8.2 software. Descriptive analyses were carried out on GPs' use of the consultations, collaborative relationships developed, GPs' satisfaction and patient care delivery. To exclude a memory bias for results, comparative analyses were carried out to ascertain that the time lapse between the consultation and the interview did not change results. As appropriate, the chi-square test was used for categorical variables and ANOVA tests for continuous variables. A 5% p level of significance was chosen.

## Results

118 GPs (response rate: 84.9%) responded, giving information for 181 patients (response rate: 69.6%). 3 GPs refused to answer. 10 GPs had moved from the area or had retired at the time of the survey. 8 GPs were not reached. Among the 181 patients, 167 (92.3%) were still being seen by the GP at the time of the survey.

### 1. Shared mental health intervention (patient profiles and management implemented within the intervention)

Patients were 77.5% female, mean age 39.7 years (sd = 14.4). 60.6% had a current professional activity. According to the main CIM-X diagnoses established by the psychiatrist at the time of the intervention, 60.4% of patients presented a mood disorder, 14.6% an anxiety disorder, 18.1% a personality disorder and 6.9% another type of disorder. Comorbidity was noted for 28.7%.

GPs referred 64.7% of the patient population concerned by the intervention (30.9% came of their own accord or were referred by their family and for 4.3%, data were not available). Among patients referred to the consultation-liaison system by GPs, 84.8% were referred because of diagnosis or therapeutic difficulties, 9.3% on patient or family demand and 5.9% for a specialized follow-up. GPs reported that in some cases it was a long time before the patient agreed to consult, and that knowing that a consultation was offered without actual transfer to the specialist system convinced certain reluctant patients.

Management implemented within the intervention was mainly collaborative, entailing dealings between GP and psychiatrists for 73.9% of patients, or for 18.5% no contact, but where this was understood by the GP (for instance when patients for personal reasons did not wish for it). GPs reported, and deplored, a lack of these collaborative relationships for 7.6% of patients. Collaborative dealings were more frequent for patients referred to the system by GPs than when patients themselves resorted to this consultation (p < .001).

### 2. Long term opinions on the shared mental health care intervention

Regarding GPs' long-term opinions on the intervention, most GPs were satisfied with the intervention (table 1).

**Table 1:** Long term GP opinions and care delivery after the consultation-liaison (23.8 months on average, N = 118 GPs and 181 patients)

**Long term GP opinions on the impact of the intervention (%)**	
GPs' opinions on the impact for primary care management (several answers possible)	

Technical management assistance	31.2
Support assistance	51.2
Third party role	10.4
Negative impact	8.8

GPs' opinions on patient medical outcome	

Positive outcome	66.8
No change	31.7
Negative outcome	1.5

**Long-term care delivery: main care provider (%)**	

GP	79.7
alone	60.4
with psychologist	10.9
with another professional	8.2
Psychiatrist (public or private practice)	20.3

GPs' opinions on patient medical outcome

The intervention provided technical management assistance for a third of patients, via changes in GPs' management (change in medication, psychotherapy orientation). According to the GPs, the main impact of the intervention was to provide support assistance for half of the patients, backing up GP management in case of patient reluctance towards treatment for example, or in case of doubt on the appropriate care on the part of the GP. The usefulness of a third party in the relationship was highlighted for 10.4% patients. GPs mentioned negative consequences for 8.8% patients, such as a feeling of "dispossession", disagreement between GP and mental health specialist, or confusing numbers of care-givers for the patient and for the GP.

Regarding patient medical outcome, GPs reported a positive outcome for more than half of the patients and no change for a third.

### 3. Long term care delivery: secondary referral rate

After the intervention, GPs remained the care providers for the psychiatric management for eight referred patients out of ten (table 1). The consultation-liaison was re-applied for 4.9% but knowing that it remained a possibility was helpful for most GPs. Classic psychiatric referral occurred for 20.3% of the patients.

## Discussion

This paper aimed to evaluate the impact of a specific collaboration intervention in real-world practice, focusing on its main users, GPs. Its strength is that it provides data from GPs on the impact of the consultation-liaison system after sufficient time lapse, incorporating, for most patients, information about how practicable the specialized advice was and whether the GP and the patient finally benefited from it or not (long-term opinions were based, for 72% of patients, on recent consultations, since only 18% were not seen during the period following the intervention). A design of this type is more powerful than a study asking these questions immediately after the intervention. The patient population was derived from a primary care population, and included anxiety disorders and personality disorders that are underrepresented in the literature [4,8-12].

### Limitations

Caution is required in the interpretation of a descriptive study based on GPs' responses collected through personal interviews, and reflecting individuals' subjective experiences, even though a high response rate was registered. The reliability and validity of a telephone questionnaire might also be questionable.

An important limitation requires discussion, and that is the particular nature of a retrospective study, the relevance of which could be more debatable because time lapses differed according to patients. This implies a possible memory bias, since recall could differ according to the time lapse between the consultation and the interview of the GP (with a median time lapse of 23.9 months). However it was possible to ascertain that this effect not significant for this survey, by implementing a t-test to determine whether the time lapse parameter differed in its relationship with the main indicators (long-term positive opinions or not, long-term secondary referral or not) and with the main confounders (patients still seen by the GP or lost, patients referred to the system by the GP or not, collaborating relationships established or not). Results were non significant.

Finally, and more seriously, the assessment focused only GPs' opinions and was not completed by independent assessments, blind to the intervention, nor by objective measurements (these may not been linked, as shown for GPs' knowledge and management of mental disorders) [5,13,14].

### Long term opinions on the shared mental health care intervention

GPs made use of the intervention, referring 65% of patients to the system, which is much more than the GP referral percentage found before the Mental Health Organizational Intervention for traditional psychiatrist consultation (25.1%) [15].

GPs' satisfaction with collaborative programs is known to be high [2,16,17]. The study explored the helpfulness of shared mental health care according to GPs, who had highlighted a need for support in a prior survey [15]. It is noteworthy that GPs reported technical management assistance for only a third of the patients and evaluated the impact of the intervention mainly as support assistance without fundamental changes in GPs' management. It is as if an intervention of this nature provides more a form of "emotional" support for complicated patients that GPs are managing in primary care, than technical management assistance.

Results, based on opinions of the GPs, have thus confirmed the benefit for primary care management and for patient medical outcome that has also been reported in the literature. The study adds to the literature by reporting positive opinions on the system and on patient outcome, even when some time had elapsed since GPs had actually referred the patients, suggesting that this mode of care is able to create a more collegiate and collaborative relationship between primary care providers and the "formal" mental health care system [6,17-20].

### Long term care delivery after the shared mental health care intervention

The main result of the study confirms the hypothesis: the GP remained the primary care provider at a distance of two years from the start of the consultation-liaison intervention. The main service delivery pattern was thus primary care, in conjunction with the support of the consultation-liaison staff, who could be required to re-intervene but who actually intervened a second time or more for less than 5% of patients. By adding the proportion of patients referred secondarily just after the consultation (30.0%) and those referred at a later date (20.3%, i.e 14.2% of the initial group of 70% of patients), it can be considered that secondary referral occurred in 44.2% of cases. This proportion is coherent with the literature (18–44%). But in our survey, for the first time, a two-year average time lapse was ensured four years on from the start of the intervention, and this time lapse is sufficient to conclude that GPs' de-motivation shown in some research into collaborative mental health care has not been confirmed here [2,17,20,21]. Further studies are still required regarding the appropriateness of the care provider.

## Conclusion

The intervention supported GP partners in their management of patients with common mental health problems. Further studies are required on the appropriateness of the care provider.

## Abbreviations

GP: General Practitioner; GPs: General Practitioners; CIM-X: International classification of diseases.

## Competing interests

The authors declare that they have no competing interests.

## Authors' contributions

NY conceived the study, participated in its design and in the acquisition of data, performed the statistical analysis and drafted the manuscript. CP drafted the manuscript. M–CH–B conceived the study and participated in its design. BF provided methodological and statistical expertise. IG participated in the interpretation of the data. All authors read and approved the final manuscript.

## Pre-publication history

The pre-publication history for this paper can be accessed here:


